# The growth in newspaper coverage of tobacco control in China, 2000-2010

**DOI:** 10.1186/1471-2458-12-160

**Published:** 2012-03-07

**Authors:** Junling Gao, Simon Chapman, Shaojing Sun, Hua Fu, Pinpin Zheng

**Affiliations:** 1 School of Public Health, Fudan University, Key Laboratory of Public Health Safety, Ministry of Education, Shanghai 200032, China; 2 Sydney School of Public Health A27, University of Sydney, NSW 2006, Australia; 3 Fudan Journalism School, Fudan University, Shanghai 200433, China

**Keywords:** Newspapers, Coverage, Tobacco control

## Abstract

**Background:**

Media coverage of tobacco-related issues can potentially shape individual beliefs, attitudes and behaviors about tobacco use. This study aims to describe news coverage of tobacco control related issues in Chinese newspapers from 2000 to 2010.

**Methods:**

All 1149 articles related to tobacco control were extracted from the Database of Chinese Important Newspapers and content analyzed for the period Jan 1, 2000 to Dec 31, 2010. The changing pattern of tobacco control topic, article type, viewpoint, and article origin, and their relationship were analysed.

**Results:**

News coverage of tobacco control related issues increased significantly (*p *< 0.01) from 2000 to 2010, with news coverage being relatively intensive in May and June (*p *< 0.01), around World No Tobacco Day. 24.9% (n = 286) of all articles focused on secondhand smoke, 25.3% (n = 291) warned about the dangers of active smoking, and 10.0% (n = 115) focused on prevention and cessation programs and campaigns. Tobacco control topics varied significantly between national vs city/regional newspapers (*χ*^2 ^= 24.09, *p *= 0.002) and article types (*χ*^2 ^= 193.35, *p *< 0.001). Articles in national newspapers had more coverage of the dangers of tobacco and on enforcing bans on tobacco-advertising. News stories centered around monitoring tobacco use and smoke free activity, while editorials focused on enforcing bans on tobacco-advertising, youth access and programs and campaigns. Letters to editors focused on the dangers of smoking, raising tax, and smoking cessation. More articles (50.4%) took an anti-tobacco position (compared with 10.5% which were pro-smoking), with the amount of negative coverage growing significantly across the decade. National articles tended to lean toward anti-tobacco, however, local articles tended mix of pro-tobacco and neutral/balance positions. Editorials seemed to be more anti-tobacco oriented, but letters to the editor tended to show a mix of anti-tobacco and pro-tobacco positions.

**Conclusion:**

Chinese newspapers are giving increasing attention to tobacco control, but coverage remains lower than in the USA and Australia. Health workers need to give higher priority to efforts to increase news coverage beyond the present concentration around World No Tobacco Day and to develop strategies for making tobacco control issues more newsworthy to both national and local news outlets.

## Background

Media coverage of tobacco-related issues can potentially shape individual beliefs, attitudes and behaviors about tobacco use [1]. Recent studies of tobacco control policy-making in several countries have demonstrated that mass media play an indispensable role in advocacy and support for law reform in tobacco control [2-6]. Efforts to increase news media coverage of both personally relevant and policy-oriented matters have therefore become a key part of comprehensive tobacco control programs [7].

With the world's largest population, China has 52.4% of adult men and 3.4% of adult women smoking [8,9]. Although the Framework Convention on Tobacco Control (FCTC) has been in force in China since 2006, there was no national law prohibiting tobacco use in public settings until 2010. As of 2006, about 46% of Chinese cities had introduced laws on tobacco control [10]. Across the country, there are more than 800 tobacco cessation clinics, although little information is available about their utilization or cessation outcomes. In addition, a growing number of activities are underway to implement smoke-free hospitals, schools and communities. Under the encouragement of the central government, some provincial and city governments have passed local regulations to strengthen tobacco control in public places such as public transport, cinemas and hospitals.

Liu et al. reported in a letter that news coverage of tobacco issues in Chinese newspapers increased dramatically in recent years, from 43 articles in 2000 to 409 articles in 2008 [11]. However, their analysis did not take into account the growing number of newspapers included in the database in noting the reported increase, reported no coding reliability coefficient and also did not assess the "slant" of articles as to whether they were in support of or opposed to tobacco control.

This study sought to describe the changing pattern of newspaper coverage of tobacco control issues in China over a decade, including the principal issues relevant to tobacco control which were reported and the dominant viewpoints or slant on tobacco control in this coverage.

## Methods

The Database of Chinese Important Newspapers http://www.cnki.net has indexed major Chinese newspapers since 2000. The database covered more than 500 newspapers, 7.95 million papers, cover 168 topics and 3600 subgroup until December 31, 2010. But not all newspapers were indexed in 2000; the number of newspapers indexed in the database has increased continually. So the increase of newspapers indexed in the database may confound the analysis of the growth of newspaper coverage. We therefore only searched news articles from 185 newspapers indexed continuously from January 1, 2000 to December 31, 2010(See additional file 1). The search keywords were fixed as 'xiyan'(smoking) OR 'xiangyan'(cigarettes) OR 'yancao'(tobacco) OR 'kong yan' (tobacco control), OR 'wu yan' (tobacco free) OR 'jin yan' (tobacco prohibition) with Chinese and English expansion. Each article obtained from the database was scrutinized to ensure that it related to tobacco control, and all without tobacco control related content were excluded. A total of 1149 articles published in 102 continually published newspapers were thus located and analyzed. This compared with Liu et al's 152 national and 363 local newspapers indexed at any point in their 2000-June 2008 study [11].

### Coding

Each article was coded for the newspaper in which it was published and date of publication (year and month), and front page (whether - yes/no -- the article was published on the front page in the newspaper). Two reviewers (JLG and PPZ) read each article and coded for the following variables: tobacco control topic, type of article, and point or view (or slant) regarding tobacco control.

To ensure reliability of the coding process, we selected a 10% random subsample (115 articles) to assess the inter-coder reliability using the Kappa statistic. The median value of κ across the coded variables was 0.96 (tobacco control topic: 0.97, type of article: 0.98, point of view: 0.93 respectively), indicating high inter-coder reliability [12]. In the case of disagreement between the two coders, a third coder was used to help make the final decision.

### Tobacco control topics

Tobacco control topics were coded to reflect the six WHO MPOWER categories [13], as well as three additional categories used in previous studies [14,15]. The topics consisted of the following:

(1) Monitoring: articles about tobacco use including (i) prevalence of tobacco use; (ii) impact of policy interventions on use; and (iii) tobacco industry marketing, promotion and lobbying;

(2) Protection: articles about protecting people from secondhand tobacco smoke, such as the establishment of regulations prohibiting smoking in public places, on public transport, in schools and hospitals;

(3) Offering help: articles on (i) smoking cessation including primary health-care services; quit lines; pharmacological therapy;

(4) Warning: articles focused on the dangers of tobacco use;

(5) Enforcement, enforce bans on tobacco advertising, promotion and sponsorship;

(6) Raising taxes: articles on tobacco

(7) Youth access, purchase and possession;

(8) Large scale smoke free activities, such as the 2008 smoke-free Olympics, the 2010 smoke-free World Expo, and the 20010 smoke-free Asian Games.

(9) Miscellaneous items which do not match any of the aforementioned topics.

### Type of article

Articles were classified into three types [4,16]: (1) news stories (a factual account of an event or issue); (2) editorials (a nonfactual account = opinion of an event or issue written by newspaper staff); (3) letters to the editor (usually written to the newspaper by a member of the community).

### Point of view

Point of view-or slant -- which denotes the overall tone of the article [5,17], was categorized into: (1) Pro-tobacco Articles that would be understood by such a person as conveying a view of smoking as in some way positive, relatively unimportant compared to other issues, or which were critical of laws, policies and individuals advocating restricting or opposing smoking; (2) Anti-tobacco Articles judged as being likely to be read by a person favorably disposed toward smoking control, as enhancing the general view that smoking and those institutions, interests, laws and policies that support it are undesirable; (3) Neutral/balanced. A neutral or balanced article was one either made no value judgments about tobacco use or control, or else provided a clear balancing of such judgments.

### Origin of article

We coded articles as being either national or local in origin. Specifically, we distinguished the origin of an article by coding whether the story was covered by a national newspaper or wire service (e.g., People's Daily, Xinhua News Agency) or by a local newspaper (e.g., Beijing daily, Guangdong Daily).

An example of coding:

September 02, 2010 Source: People's Daily Page:8

*"No smoking" law comes into effect in south China's Guangzhou*

A local smoking-control law came into effect Wednesday in Guangzhou, the capital of south China's Guangdong Province. The law covers all of Guangzhou City. There, smoking in most public places, like offices, conference rooms, halls and elevators, is strictly prohibited. Places of business larger than 150 square meters or having more than 75 seats may designate an area for smokers. Those who break the law will be fined 50 yuan (about 7.35

*U.S. dollars) and businesses not meeting their obligations will be fined up to 30,000 yuan*.

*However, some doubt the law is enforceable*.

......

*"A quick smoke can take less than one minute. How can an enforcement agency come in such little time?" a different pedestrian asked a Xinhua reporter*.

*Hu Angang, an economics professor at Tsinghua University, said....... "Putting goals into the national plan shows great political will from state leaders. So the whole of society, especially local governments, will implement it without hesitation. The plan is a binding document for the government," said Hu*.

*Shanghai began implementing a regulation to ban smoking in 12 types of public places March 1 in an effort to have a smoke-free World Expo*.

......

*One of the world's largest tobacco producing and consuming nations, China manufactures about 100 billion packets of cigarettes each year*.

The article was published in People's Daily on September 02, 2010, and only reported the local smoking-control law in Guangzhou without making value judgments about tobacco use or control, so it was coded as:

Tobacco control topic: protection

Type of article: news stories

Point of view: neutral/balanced

Origin of article: national article

Year: 2010

Month: September

Front page: No

### Statistical analysis

The analyses reported here are based on the relative frequency of articles within each of the coded variables. We used chi-square analysis to determine associations between categorical variables as appropriate. Where there were significant associations we examined a contingency table including standardized residuals to determine what was driving the significant result. Standardized residuals convert the difference between the observed value and expected value into a z-score. Standardized residuals larger than two, therefore, indicate large differences between observed values and expected values that drive a significant chi-square result [2]. In addition, Poisson heterogeneity tests were used to test whether the number of articles in each year was the same [18].

## Results

**Figure 1:**
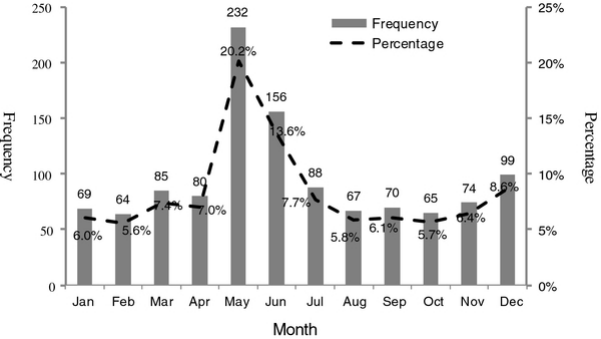
**Distribution of newspaper articles from Jan to Dec in Chinese newspapers. *P *= Percentage**.

A total of 1149 articles related to tobacco control were located in the 102 newspapers tracked across the decade. There was a statistically significant difference in the number of articles appearing each year (*p *< 0.001). From 2000 to 2004, an average of just 36 articles was published across all newspapers every year. In 2005, the number reached 61, and grew continuously until 2008, when 209 were published. 2009 saw a small fall (n = 191). And in 2010, 271 articles were published (Table 1). Similarly, there was a significant difference in the number of articles appearing each month (*p *< 0.001). More than 20% of all articles (20.2%, 232 articles) were published in May, and 13.6% (156 articles) were published in June, both being higher than in other months (Figure 1).

**Table 1:** Policy related to tobacco control from 2000 to 2010 in China [n(%)]

Policy type	2000	2001	2002	2003	2004	2005	2006	2007	2008	2009	2010	Total
Monitoring	2(1.6)	0(0)	1(0.8)	2(1.6)	3(2.4)	3(2.4)	10(7.9)	20(15.9)	24(19.0)	17(13.5)	44(34.9)	126(11.0) *

Protection	7(2.4)	1(0.3)	2(0.7)	6(2.1)	2(0.7)	12(4.2)	11(3.8)	36(12.6)	62(21.7)	55(19.2)	92(32.2)	286(24.9) *

Offering help	0(0)	0(0)	0(0)	2(3.7)	1(1.9)	2(3.7)	7(13.0)	6(11.1)	15(27.8)	10(18.5)	11(20.4)	54(4.7) *

Warning	22(7.6)	18(6.2)	15(5.2)	22(7.6)	18(6.2)	24(8.2)	36(12.4)	34(11.7)	37(12.7)	45(15.5)	20(6.9)	291(25.3) *

Enforcement	3(2.9)	6(5.8)	3(2.9)	5(4.8)	5(4.8)	5(4.8)	5(4.8)	11(10.6)	6(5.8)	14(13.5)	41(39.4)	104(9.1) *

Raising taxes	0(0)	1(1.6)	1(1.6)	0(0)	1(1.6)	0(0)	6(9.5)	2(3.2)	14(22.2)	24(38.1)	14(22.2)	63(5.5) *

Youth access, purchase and possession	2(3.0)	1(1.5)	5(7.5)	3(4.5)	7(10.4)	10(14.9)	5(7.5)	9(13.4)	11(16.4)	5(7.5)	9(13.4)	67(5.8) *

Large scale smoke free activity	0(0)	0(0)	0(0)	0(0)	1(2.3)	0(0)	2(4.7)	9(20.9)	20(46.5)	2(4.7)	9(20.9)	43(3.7) *

Miscellaneous	2(1.7)	3(2.6)	4(3.5)	3(2.6)	2(1.7)	5(4.3)	8(7.0)	18(15.7)	20(17.4)	19(16.5)	31(27.0)	115(10.0) *

Total	38(3.3)	30(2.6)	31(2.7)	43(3.7)	40(3.5)	61(5.3)	90(7.8)	145(12.6)	209(18.2)	191(16.6)	271(23.6)	1149(100) *

*Poisson heterogeneity test *p *< 0.001

Overall, 24.9% (n = 286) of articles focused on protecting people from tobacco smoke, 25.3% (n = 291) focused on information about the dangers of tobacco, and 10% (n = 115) focused on prevention and cessation programs and campaigns. For each topic, there was a significant difference in the number of articles appearing each year (p < 0.001). Few were published before 2005, and then numbers increased sharply. For example, only five articles about cessation were published before 2006, with this increasing to 11 (20.4%) in 2010. The first article about smoke free events (smoke-free Olympics, smoke-free World Expo, and smoke-free Asian Games) appeared in 2004, with most (46.5%) published in 2008 when the Olympic Games were held in Beijing, 20.9% appeared in 2010 when World Expo held in Shanghai (Table 1).

Overall, 908 articles (79%) featured in national newspapers and 241 (21%) were published on local newspapers. There was a significant difference in the topics between the two article origins (*χ*^2 ^= 24.094, df = 8, *p *= 0.002). So we conducted standardized residual analysis to identify what was driving this significant result. The national newspaper articles reported more on warning(27.0%) and enforcement(10.1%); less on monitoring(9.7%) and protection(23.8%), while the local articles reported more on monitoring(15.8%), protection(29.0%); less on warning(19.1%) and enforcement(5.0%).

There were 590 news stories (51.3%), 247 editorials (21.5%), 312 letters to the editor (27.2%). We found a significant association between tobacco control topic and article type (*χ*^2 ^= 190.353, df = 16, *p *< 0.001). Standardized residual analysis indicated that news stories were more likely than expected focus on monitoring (18.8%) or smoke free activity (15.9%) and less likely to focus on enforcement(5.3%); editorials were more likely than expected to focus on enforcement(15.4%), youth access(9.3%) or programs and campaigns(6.1%), and less likely to concern smoke free activity(3.6%) or monitoring(4.9%). Letters to the editor were more likely than expected to concern warning(33.0%), enforcement(8.0%) or raising taxes(9.0%), and less likely to concern monitoring(1.0%) or programs and campaigns(0.6%). There were only 77 articles (6.7%) published on the front page. These focused on protection (37.7%) and monitoring (15.6%) and warning (13.0%).

**Figure 2:**
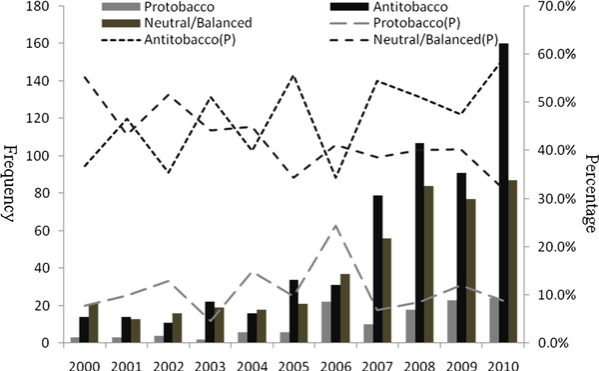
**Trends of article points from 2000 to 2010 in Chinese newspapers**.

There were 121 articles classified as pro-tobacco (10.5%), 449 neutral/balanced (39.1%) and 579 anti-tobacco (50.4%). Before 2004, the number of these three kinds of articles remained low. After 2004, anti-tobacco articles increased more sharply than pro- or neutral/balanced articles. In 2010, the number of anti-tobacco articles was twice as common as neutral/balanced articles and eight times more frequent than pro-tobacco articles (Figure 2).

**Figure 3:**
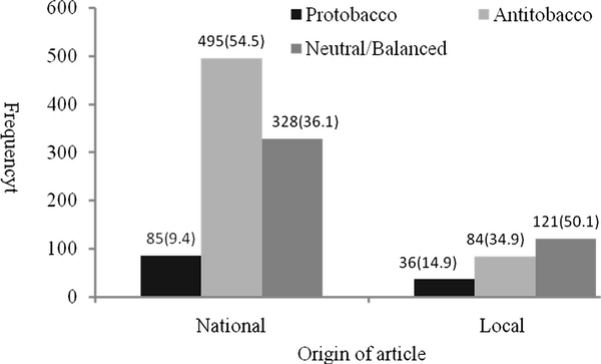
**Viewpoints of the national articles and the local articles [n(%)]**.

There was a significant association between point of view and article origin (*χ*^2 ^= 29.901, df = 2, *p *< 0.001), with national articles being more likely to express anti-tobacco views, while local articles were more likely to express pro-tobacco or neutral/balanced views (Figure 3).

**Figure 4:**
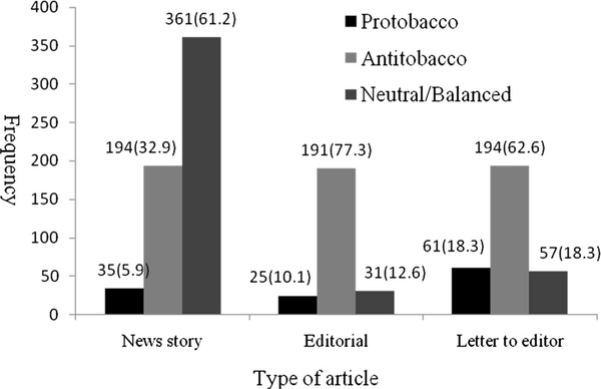
**Viewpoints of three different types of articles [n(%)]**.

Similarly, we also found a significant association between point of view and article type (*χ*^2 ^= 268.91, df = 4, *p *< 0.001). News stories were more likely to express neutral/balanced points of view; editorials were more anti-tobacco, while letters to the editor were predictably more inclined both to pro-tobacco and anti-tobacco views (Figure 4).

## Discussion

News coverage plays a key role in shaping public and political attitudes about tobacco control [1]. Our findings suggest that Chinese newspapers paid little attention to tobacco control issues prior to 2005, but thereafter news attention increased, which was consistent with Liu's study finding [11]. Chinese newspapers are giving increasing attention to tobacco control, but coverage remains low in comparison to the USA and Australia [19-21]. There remains much potential for journalists, health workers and citizens to increase coverage of tobacco control matters in China.

Several developments were associated with the observed increase in reporting: the Chinese Government signed the WHO Framework Convention on Tobacco Control (FCTC) in 2003 and the Standing Committee of the National People's Congress ratified it in 2005; the 2008 Beijing smoke-free Olympics; the Notice on Adjusting Consumption Tax of Tobacco Products came into force on May 1, 2009 [22]; and the smoke-free World Expo was held in Shanghai in 2010. These all stimulated provincial and city governments to pass local bans prohibiting smoking in public settings, which attracted news attention. For example, the Notice on Adjusting Consumption Tax of Tobacco Products came into force in 2009, causing the number of articles about tobacco tax to reach peak (Table 1). Tobacco tax - arguably the most effective of all tobacco control strategies - is poorly covered in Chinese news media. A large population of Chinese smokers has low incomes and is likely to be price-sensitive. Future tobacco advocacy should target raising media attention to the strong positive effect of increasing tobacco tax on cessation.

As has been reported in other studies, the impact of special days focused on tobacco control [11,23,24] can generate spikes in media attention to tobacco control. However, when the year round coverage of tobacco control related issues is low as it is in China, concerns arise when journalists concentrate this coverage around just one day and publish relatively few stories during the rest of the year. Days like World No Tobacco Day can be important conduits for news attention, but they can also send an unfortunate message that attention to tobacco control is not a mainstream news area. Indeed, only 6.7% (n = 77 in 10 years) of articles were published on the front page, suggesting that tobacco control is seldom regarded by editors as a leading news issue.

Tobacco control advocates should learn how to harness the synergistic effects of national and local developments. In recent years, city development has been very fast in China. Advocates should make use of opportunities arising from large-scale city-image-building activities, and seek to encourage smoke free policies as integral to these.

It is encouraging that more than half of articles (50.4%) were anti-tobacco, with this proportion increasing across the decade. National articles were significantly more inclined to support tobacco control than were local articles. In China, national newspapers are usually sponsored by the central government, while local newspapers are sponsored by local government or their branches. Local economy protectionism may explain the discrepancy in the support shown for tobacco control between national and local media. Tobacco producers typically contribute significantly to local economies, and have close ties with local governments. Tobacco businesses may receive political support from local governments, including the publishing of stories supportive of the tobacco industry.

There are several limitations in this study. First, our analysis is limited to newspaper coverage of tobacco control issues. Newspapers are only one of many news outlets that may affect public opinion. But the coverage of issues in newspapers is typically highly correlated with the presentation of the same issues in other media such as radio and television [25], with morning newspapers often setting the agenda for coverage by other media during the day. Second, we coded tobacco control policy according to the categories of the WHO MPOWER package, which differ from previous research [4,6,15,16] and may be not suitable comparing with that research. Two recent studies [10,26] on implementation of the FCTC and MPOWER in China showed that tobacco control in China is relatively undeveloped and that there are large gaps in meeting FCTC standards. Finally, because we could not obtain the circulation of each newspaper from the database, we could not analyze how many people were reached and potentially influenced by the coverage we report.

## Conclusion

Chinese newspapers are giving increasing attention to tobacco control, but coverage remains low by international benchmarks. Health workers need to give higher priority to efforts to increase news coverage beyond the present concentration around World No Tobacco Day and to develop strategies for making tobacco control issues more newsworthy to both national and local news outlets.

## Competing interests

The authors declare that they have no competing interests.

## Authors' contributions

JG carried out the current study, participated in its design, performed the statistical analysis, and drafted the manuscript. PZ and SJ participated in study design and coded articles, and revised the manuscript critically for important intellectual content. SC and HF participated in study design and revised it critically for important intellectual content. All authors read and approved the final manuscript.

## Pre-publication history

The pre-publication history for this paper can be accessed here:

http://www.biomedcentral.com/1471-2458/12/160/prepub

## Supplementary Material

Additional file 1 **The list of continually published newspaper**. Click here for file
